# Efficient production of acetoin in *Saccharomyces cerevisiae* by disruption of 2,3-butanediol dehydrogenase and expression of NADH oxidase

**DOI:** 10.1038/srep27667

**Published:** 2016-06-09

**Authors:** Sang-Jeong Bae, Sujin Kim, Ji-Sook Hahn

**Affiliations:** 1 School of Chemical and Biological Engineering, Seoul National University, Institute of Chemical and Processes, 1 Gwanak-ro, Gwanak-gu, Seoul 08826, Republic of Korea

## Abstract

Acetoin is widely used in food and cosmetic industry as taste and fragrance enhancer. For acetoin production in this study, *Saccharomyces cerevisiae* JHY605 was used as a host strain, where the production of ethanol and glycerol was largely eliminated by deleting five alcohol dehydrogenase genes (*ADH1*, *ADH2*, *ADH3*, *ADH4*, and *ADH5*) and two glycerol 3-phosphate dehydrogenase genes (*GPD1* and *GPD2*). To improve acetoin production, acetoin biosynthetic genes from *Bacillus subtilis* encoding α-acetolactate synthase (AlsS) and α-acetolactate decarboxylase (AlsD) were overexpressed, and *BDH1* encoding butanediol dehydrogenase, which converts acetoin to 2,3-butanediol, was deleted. Furthermore, by NAD^+^ regeneration through overexpression of water-forming NADH oxidase (NoxE) from *Lactococcus lactis*, the cofactor imbalance generated during the acetoin production from glucose was successfully relieved. As a result, in fed-batch fermentation, the engineered strain JHY617-SDN produced 100.1 g/L acetoin with a yield of 0.44 g/g glucose.

Acetoin, also known as 3-hydroxy-2-butanone or acetylmethylcarbinol, is widely used in food industry as a flavor enhancer, giving a buttery taste^1^. It can also be used as a building block for various chemicals such as alkyl pyrazines, diacetyl, and acetylbutanediol^2^,^3^,^4^. Currently, most of commercial acetoin is produced by chemical synthesis, but the use of such non-natural acetoin is restricted in some applications, especially in food and cosmetic industry, because of safety concerns. Accordingly, many attempts have been reported to produce natural acetoin by biological process, including enzyme conversion and microbial fermentation^1^,^5^,^6^,^7^.

Many microorganisms, such as *Bacillus subtilis*, *Bacillus amyloliquefaciens*, *Enterobacter cloacae*, *Serratia marcescens*, and *Paenibacillus polymyxa*, can produce acetoin from pyruvate via α-acetolactate by two enzymatic steps catalyzed by α-acetolactate synthase and α-acetolactate decarboxylase^1^,^8^. Acetoin can be further converted to 2,3-butanediol by 2,3-butanediol dehydrogenase (also known as acetoin reductase) using NADH as a cofactor. Therefore, to accumulate acetoin, 2,3-butanediol production was inhibited in various bacteria mainly by adopting two strategies; disruption of 2,3-butanediol dehydrogenase and overexpression of NADH oxidase. Butanediol dehydrogenase-blocked *B. subtilis* (JNA-UD-6), isolated after mutagenesis using UV irradiation with diethyl sulfate, showed a 24.3% increase in acetoin production and a 39.8% decrease in 2,3-butanediol production compared with the parental strain in batch fermentation, and produced 53.9 g/L acetoin after 144 h fermentation in fed-batch fermentation^9^. On the other hand, NADH oxidase, which converts NADH to NAD^+^, was overexpressed to reduce NADH-dependent 2,3-butanediol production. In *S. marcescens* H32, introduction of NADH oxidase from *Lactobacillus brevis* decreased 2,3-butanediol titer by 48% and increased acetoin titer by 33%^10^. Both of these strategies have also been applied to *B. subtilis* and *E. cloacae,* resulting in 56.7 g/L and 55.2 g/L acetoin production, respectively^11^,^12^.

*Saccharomyces cerevisiae*, which is classified as generally recognized as safe (GRAS) microorganism, has been considered as a key cell factory platform for producing valuable chemicals because of its tolerance and robustness toward industrial conditions. Acetoin accumulation has been reported in commercial wine yeast engineered for glycerol overproduction^13^. The engineered strain overexpressing *GPD1* and deleting *ALD6* (BC *GPD1 ald6*) produced 26.9 g/L glycerol with 9.5 g/L acetoin from 200 g/L glucose. In addition, several efforts have been made to engineer *S. cerevisiae* to produce 2,3-butanediol, a neighboring metabolite of acetoin, leading to a significant improvement in both titer and yield of 2,3-butanediol production^14^,^15^,^16^. Previously, we monitored acetoin production levels to investigate aromatic amino acids-inducible promoter system^17^. Nevertheless, the study focused on acetoin production in *S. cerevisiae* has not yet been reported.

In our previous study, we developed *S. cerevisiae* strain for efficient production of 2,3-butanediol by introducing heterologous acetoin biosynthetic pathway from *B. subtilis*, overexpressing 2,3-butanediol dehydrogenase, and eliminating major byproduct pathways involved in ethanol and glycerol production^14^. Furthermore, the cofactor imbalance generated during 2,3-butanediol production in the engineered strain was restored by overexpressing water-forming NADH oxidase from *Lactococcus lactis*. In this study, for efficient production of acetoin, we additionally disrupted 2,3-butanediol dehydrogenase in *adh1-5*Δ*gpd1*Δ*gpd2*Δ strain and adopted above strategies comprised of introducing heterologous acetoin pathway and redox rebalancing.

## Results and Discussion

### Introduction of acetoin biosynthetic pathway in *adh1-5*Δ*gpd1*Δ*gpd2*Δ strain (JHY605)

**Figure 1:**
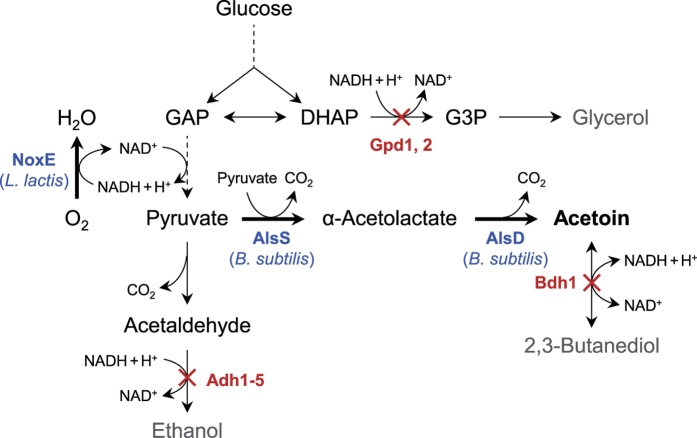
Metabolic pathway for acetoin production used in this study. Two molecules of pyruvate are converted to one molecule of acetoin by α-acetolactate synthase (AlsS) and α-acetolactate decarboxylase (AlsD). Acetoin can be further reduced to 2,3-butanediol by 2,3-butanediol dehydrogenase (Bdh1). The water-forming NADH oxidase (NoxE) catalyzes the oxidation of NADH to NAD^+^ using oxygen as an electron acceptor. Dashed arrows indicate multiple enzymatic steps. The crosses represent blockage of the pathway by deleting the corresponding genes. DHAP, dihydroxyacetone phosphate; G3P, glycerol-3-phosphate; GAP, glyceraldehyde-3-phosphate.

**Figure 2:**
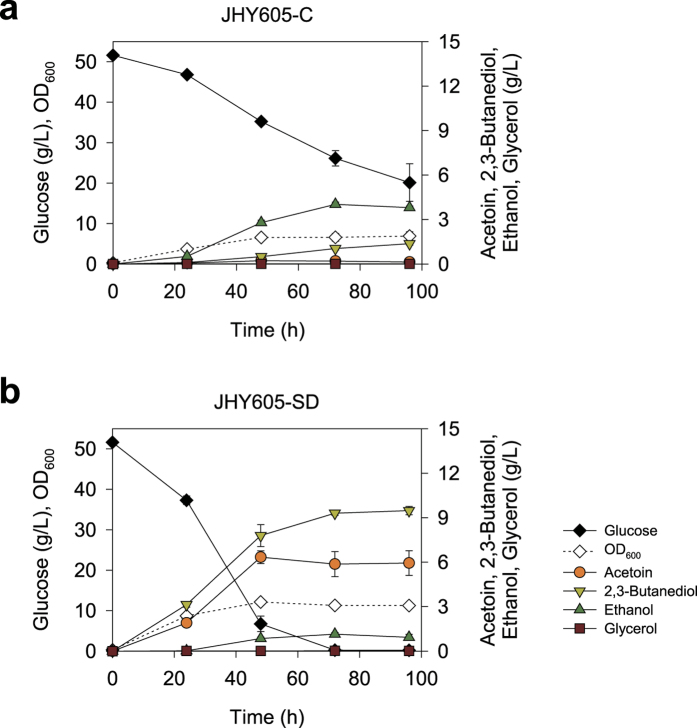
Acetoin production by introducing acetoin biosynthetic pathway. Strain JHY605 (*adh1-5*Δ*gpd1*Δ*gpd2*Δ) harboring empty p413GPD plasmid (JHY605-C) **(a)** or p413-SD (JHY605-SD) **(b)** was grown in 10 mL SC-His media containing 50 g/L glucose in a 100 mL flask. Error bars indicate standard deviations of three independent experiments.

To produce acetoin in *S. cerevisiae*, α-acetolactate synthase (*alsS*) and α-acetolactate decarboxylase (*alsD*) genes from *B. subtilis* were introduced into JHY605 strain (*adh1-5*Δ*gpd1*Δ*gpd2*Δ), which lacks five alcohol dehydrogenases (Adh1 to Adh5) and two glycerol 3-phosphate dehydrogenases (Gpd1 and Gpd2) (Fig. 1). The fermentation profiles of the control strain JHY605-C harboring empty vector p413GPD and strain JHY605-SD expressing *alsS* and *alsD* from p413-SD plasmid are shown in Fig. 2. JHY605-C produced only a trace amount of acetoin (0.1 g/L) and 1.4 g/L of 2,3-butanediol from 31.6 g/L of glucose after 96 h fermentation (Fig. 2a). Although five alcohol dehydrogenase genes were deleted, JHY605-C produced 3.8 g/L of ethanol as a major end product. This might be because pyruvate generated by glycolysis is mainly metabolized to ethanol production via pyruvate decarboxylases and remaining ADH isozymes including Sfa1, Adh6, and Adh7. In agreement with previous study, glycerol pathway was completely blocked in JHY605-C by the deletion of *GPD1* and *GPD2*^14^,^18^.

Whereas, strain JHY605 harboring p413-SD plasmid (JHY605-SD) showed an increase in glucose consumption rate and produced up to 5.9 g/L acetoin (Fig. 2b). Furthermore, 2,3-butanediol production level increased to 9.3 g/L, even though 2,3-butanediol dehydrogenase was not overexpressed, reflecting the endogenous 2,3-butanediol dehydrogenase activity in *S. cerevisiae*. By introducing this competing pathway, ethanol production yield was reduced to 0.02 g/g glucose in JHY605-SD compared with that in JHY605-C (0.12 g/g glucose).

In *S. cerevisiae*, NAD^+^ regeneration for glycolysis is mainly achieved by producing ethanol and glycerol (Fig. 1). Therefore, the growth defects of strain JHY605 might be due to the accumulation of NADH. In addition, the residual ADH activity might not be enough to prevent the accumulation of toxic acetaldehyde (Fig. 1). Introduction of acetoin biosynthetic pathway into JHY605 might relieve the growth defects by reducing acetaldehyde formation through efficient conversion of pyruvate to acetoin, and also by partly restoring NAD^+^ regeneration through 2,3-butanediol production (Fig. 1).

### Disruption of 2,3-butanediol dehydrogenase *BDH1* to improve acetoin production

**Figure 3:**
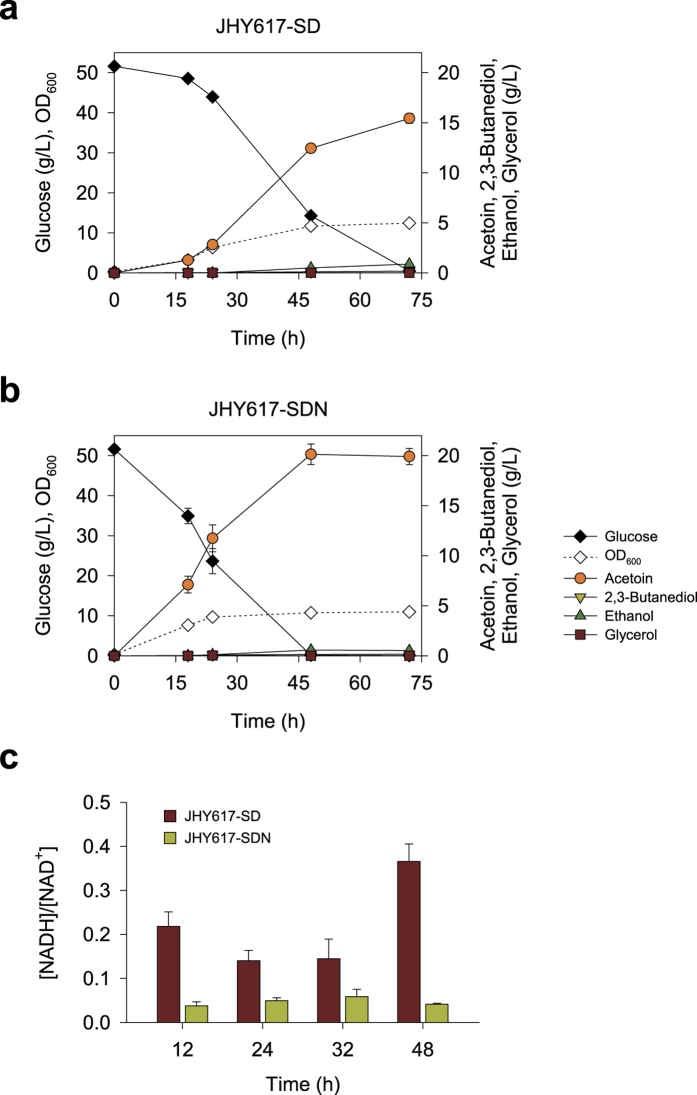
Improvement of acetoin production by deleting *BDH1* and by expressing NADH oxidase (NoxE). Strain JHY617 (*adh1-5*Δ*gpd1*Δ*gpd2*Δ*bdh1*Δ) harboring p413-SD (JHY617-SD) **(a)** or p413-SDN (JHY617-SDN) **(b)** was grown in 10 mL SC-His containing 50 g/L glucose in a 100 mL flask. **(c)** NADH/NAD^+^ ratios in JHY617-SD and JHY617-SDN. Error bars indicate standard deviations of three independent experiments.

By introducing acetoin biosynthetic pathway into JHY605, pyruvate flux was successfully redirected toward acetoin pathway. However, acetoin was further converted to 2,3-butanediol, resulting in about 1.5-fold higher titer of 2,3-butanediol than that of acetoin. In *S. cerevisiae*, Bdh1 is a major enzyme catalyzing the reduction of acetoin to 2,3-butanediol (Fig. 1). Therefore, we further deleted *BDH1* gene in JHY605, resulting in strain JHY617. When acetoin biosynthetic pathway was introduced into strain JHY617 (JHY617-SD), 2,3-butanediol production from acetoin was significantly reduced to 0.2 g/L, which then contributed to the increase in acetoin production accordingly. As a result, up to 15.4 g/L acetoin was produced after 72 h fermentation in SC-His medium containing 50 g/L glucose, with a yield of 0.30 g/g glucose (Fig. 3a). The trace amount of 2,3-butanediol production in JHY617-SD might be mediated by other minor putative enzymes such as D-arabinose dehydrogenase (Ara1) having 2,3-butanediol dehydrogenase activity^19^.

### Recovering redox imbalance by expressing water-forming NADH oxidase *noxE*

Cofactor balance, especially NADH/NAD^+^ ratio plays an important role in a large number of biochemical reactions^20^,^21^. Thus, maintaining the cofactor balance is an essential requirement for sustaining cellular metabolism and cell growth^22^. In acetoin production pathway, NADH produced from glycolysis could not be converted to NAD^+^, leading to a redox cofactor imbalance. Furthermore, since NADH-dependent metabolic pathways, related to the production of ethanol, glycerol, and 2,3-butanediol, were disrupted in strain JHY617-SD, the redox imbalance might be more severe. Therefore, as an effort to resolve the redox imbalance in JHY617-SD, we introduced *noxE* from *L. lactis*, encoding water-forming NADH oxidase. To this end, *FBA1* promoter controlled-*noxE* was inserted to the acetoin biosynthetic plasmid p413-SD, resulting in p413-SDN. Strain JHY617 harboring p413-SDN (JHY617-SDN) showed a significant improvement in glucose consumption rate, thereby taking less time (~48 h) to completely ferment 50 g/L glucose than it was taken for JHY617-SD (~72 h) (Fig. 3). Moreover, acetoin production was improved up to 20.1 g/L with a yield of 0.39 g/g glucose, reaching 80% of maximum theoretical yield. Accordingly, strain JHY617-SDN exhibited about two-fold increase in acetoin productivity (0.42 g/(L·h)) compared with JHY617-SD (0.21 g/(L·h)), suggesting that redox imbalance caused by acetoin production was successfully alleviated by expressing NADH oxidase (Table 1). To confirm the effect of *noxE* expression on redox state, we analyzed intracellular NADH/NAD^+^ ratios in JHY617-SD and JHY617-SDN. As expected, the NADH/NAD^+^ ratios in JHY617-SDN were lower than those in JHY617-SD throughout the growth phase, demonstrating the efficient conversion of NADH to NAD^+^ by NoxE (Fig. 3c).

### Fed-batch fermentation for acetoin production

**Figure 4:**
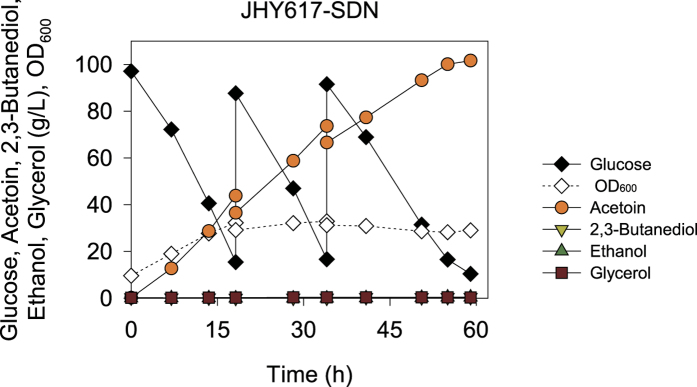
Fed-batch fermentation of JHY617-SDN for acetoin production. Strain JHY617-SDN was cultivated in YPD medium containing 100 g/L glucose with initial OD_600 _of 9.5. Glucose was intermittently added into culture medium using the feeding solution (800 g/L glucose) before glucose was completely consumed.

To evaluate the potential of JHY617-SDN as a host strain for acetoin production, fed-batch fermentation was performed with intermittent feeding of glucose and pH control. JHY617-SDN was grown in YPD medium containing 100 g/L glucose with initial OD_600_ of 9.5. In fed-batch fermentation, up to 100.1 g/L acetoin was produced with a yield of 0.44 g/g glucose after 55 h cultivation, reaching 90% of maximum theoretical yield (Fig. 4). Moreover, acetoin productivity was dramatically improved to 1.82 g/(L·h). Taken together, JHY617-SDN showed superior performance of acetoin production compared with the host strains reported in previous studies (Table 2). Notably, both acetoin titer and yield in this study are the highest among these studies. Although acetoin productivity reported in *S. marcescens* and *E. cloacae* were higher than that of our study^10^,^12^, these strains have potential pathogenicity^23^,^24^.

In this study, we developed *S. cerevisiae* strain for efficient production of acetoin by introducing heterologous acetoin pathway from *B. subtilis* and eliminating 2,3-butanediol dehydrogenase using JHY605 as a host strain, where the production of ethanol and glycerol was largely eliminated. In addition, cofactor imbalance generated during acetoin production was successfully alleviated by expressing NADH oxidase from *L. lactis*, leading to significantly enhanced acetoin production. As a result, to the best of our knowledge, the highest titer and yield in microbial production of acetoin were achieved. These results suggest that *S. cerevisiae* might be a promising host for the production of acetoin.

## Methods

### Strains and media

All strains used in this study are described in Table 3. JHY617 strain, a *BDH1* deletion mutant derived from JHY605^14^, was generated by PCR-mediated homologous recombination. The *bdh1*Δ*::KanMX6* cassette flanked by 300 bp upstream and 282 bp downstream of the *BDH1* open reading frame was obtained by PCR amplification from genomic DNA of *bdh1*Δ strain (BY4741 *bdh1*Δ*::KanMX6*, EUROSCARF) as a template, using the primer pair of d_BDH1 F (5′-GATTTGCTCACGCTACTTTG-3′) and d_BDH1 R (5′-GCCATGCTTTGTTTTAGACG-3′). The resulting PCR product was transformed into JHY605 strain and transformants were selected on YPD plate (10 g/L yeast extract, 20 g/L bacto-peptone, and 20 g/L glucose) supplemented with 200 μg/mL G418 sulfate (AG Scientific, Inc.)

Yeast cells were cultured in YPD medium or in synthetic complete medium lacking histidine (SC-His) (20 or 50 g/L glucose, 6.7 g/L yeast nitrogen base without amino acids, and 1.92 g/L amino acids mixture lacking histidine).

### Plasmid construction

Plasmids used in this study are described in Table 4. The recombinant plasmids for acetoin pathway were constructed by using the multiple cloning system as previously described with minor modifications^14^. The *alsS*-expression cassette (P_*TDH3*_-*alsS*-T_*CYC1*_) flanked by MauBI and and NotI sites was obtained by PCR from p413_P_*TDH3*_-*alsS*-T_*CYC1*_ using the primers, Univ F2 (5′-GACTCGCGCGCGGGAACAAAAGCTGGAGCTC-3′) and Univ R (5′-GACTACGCGTGCGGCCGCTAATGGCGCGCCATAGGGCGAATTGGGTACC-3′), and cloned into AscI and NotI sites of p413-D plasmid^14^, resulting in p413-SD. The *noxE*-expression cassette (P_*FBA1*_-*noxE*-T_*FBA1*_) was additionally cloned into p413-SD as previously described^14^, resulting in p413-SDN.

### Fermentation conditions

For flask fermentation, yeast cells harboring appropriate plasmids were pre-cultured in 5 mL of SC-His medium containing 20 g/L glucose in a 50 mL flask, inoculated to OD_600 _of 0.3 in 10 mL of SC-His medium containing 50 g/L glucose in a 100 mL flask, and then cultivated at 30 °C with shaking at 170 rpm.

Fed-batch fermentation was performed in 500 mL YPD medium containing 100 g/L glucose using a 1 L bench-top fermenter FMT-DS (Fermentec, Korea) at 30 °C with agitation speed of 500 rpm and air flow rate of 1.0 vvm. The pH of the culture medium was maintained at 5.5 by using 4 N NaOH. Strain JHY617-SDN was pre-cultured in SC-His medium containing 20 g/L glucose and inoculated into the fermenter with initial OD_600_ of 9.5. The feeding solution (800 g/L glucose) was intermittently added to the culture medium when the glucose concentration was lower than 20 g/L.

### Analytical methods

Cell growth was determined by measuring the optical density at 600 nm (OD_600_). To analyze profile of metabolites, 1 mL of culture supernatants were collected and filtered through a 0.22 μm syringe filter. The concentrations of glucose, glycerol, acetoin, 2,3-butanediol, and ethanol were determined by high performance liquid chromatography (HPLC) using UltiMate 3000 HPLC system (Thermo fishers scientific) equipped with a BioRad Aminex HPX-87H column (300 mm×7.8 mm, 5 μm, Bio-rad) at 60 °C with 5 mM H_2_SO_4_ at a flow rate of 0.6 mL/min and refractive index (RI) detector at 35 °C. The intracellular concentrations of NADH and NAD^+^ were measured using EnzyChrom™ NAD/NADH Assay Kit (E2ND-100, BioAssay Systems). Strains JHY617-SD and JHY617-SDN were harvested at different time points of fermentation and washed with cold phosphate-buffered saline (PBS; 8 g/L NaCl, 0.2 g/L KCl, 1.42 g/L Na_2_HPO_4_, 0.24 g/L KH_2_PO_4_ [pH 7.4]) solution. Cells of OD_600_ of 1.0 were pelleted and analyzed according to the manufacturer’s instructions.

## Additional Information

**How to cite this article**: Bae, S.-J. *et al*. Efficient production of acetoin in *Saccharomyces cerevisiae* by disruption of 2,3-butanediol dehydrogenase and expression of NADH oxidase. *Sci. Rep.* **6**, 27667; doi: 10.1038/srep27667 (2016).

## Figures and Tables

**Table 1:** Fermentation characteristics of recombinant strains.

**Strain**	**Description**	**Fermentation time (h)**	**Cell density (OD**_**600**_)	**Consumed glucose (g/L)**	**Products (g/L)**				**Productivity of acetoin (g/(L·h))**	**Yield of acetoin (g/g glucose)**
					**Ethanol**	**Glycerol**	**2,3-BDO**	**Acetoin**		
Batch flask fermentation in SC-His medium										
JHY605-C	*adh1-5*Δ*gpd1*Δ*gpd2*Δ [EV]	96	6.89 ± 1.07	31.6 ± 4.64	3.8 ± 0.12	0.01 ± 0.00	1.37 ± 0.10	0.13 ± 0.02	0.001 ± 0.000	0.004 ± 0.000
JHY605-SD	*adh1-5*Δ*gpd1*Δ*gpd2*Δ [SD]	72	11.29 ± 0.15	51.5 ± 0.16	1.14 ± 0.12	0.02 ± 0.00	9.30 ± 0.14	5.87 ± 0.84	0.082 ± 0.012	0.114 ± 0.016
JHY617-SD	*adh1-5*Δ*gpd1*Δ*gpd2*Δ*bdh1*Δ [SD]	72	12.42 ± 0.50	51.1 ± 0.04	0.89 ± 0.06	0.01 ± 0.00	0.18 ± 0.16	15.43 ± 0.49	0.214 ± 0.007	0.302 ± 0.007
JHY617-SDN	*adh1-5*Δ*gpd1*Δ*gpd2*Δ*bdh1*Δ [SDN]	48	10.76 ± 0.20	51.5 ± 0.04	0.58 ± 0.13	0.01 ± 0.00	0.14 ± 0.01	20.13 ± 1.02	0.419 ± 0.021	0.391 ± 0.020
Fed-batch fermentation in YPD medium										
JHY617-SDN	*adh1-5*Δ*gpd1*Δ*gpd2*Δ*bdh1*Δ [SDN]	55	28.25	227.7	0.40	0.34	0.39	100.08	1.820	0.439

**Table 2:** Comparison of acetoin production by various microorganisms.

**Strains**	**Carbon source**	**Culture condition**	**Description**	**Titer (g/L)**	**Productivity (g/(L·h))**	**Yield (%)**	**Reference**
Bacteria							
*B. subtilis*	Glucose	Batch	Isolated from sea sediment Optimization of medium components and culture conditions	76.0	1.00	74.0	25
*B. subtilis*	Glucose	Fed-batch	Overexpression of BDH Two-stage pH control strategy	73.6	0.77	83.6	26
*B. subtilis*	Glucose	Batch	Inactivation of BDH Screening and expression of NADH oxidase from *B. subtilis*	56.7	0.68	77.3	11
*B. amyloliquefaciens*	Glucose	Batch	Acetoin tolerant mutant by adaptive evolution Two-stage agitation speed control strategy	71.5	1.63	84.5	27
*S. marcescens*	Sucrose	Fed-batch	Expression of NADH oxidase from *L. brevis*	75.2	1.88	70.0	10
*P. polymyxa*	Glucose	Fed-batch	Isolated from orchard soil Optimization of medium components and culture conditions	55.3	1.32	75.6	28
*E. cloacae*	Glucose	Fed-batch	Inactivation of BDH and byproduct pathways Expression of NADH oxidase from *L. brevis*	55.2	2.69	76.3	12
Yeast							
*Candida glabrata*	Glucose	Batch	Inactivation of BDH and byproduct pathways Overexpression of *PDC1* Expression of NADH oxidase from *L. lactis*	7.3	0.11	14.9	6
*S. cerevisiae*	Glucose	Fed-batch	Inactivation of BDH and byproduct pathways Introduction of acetoin pathway from *B. subtilis* Expression of NADH oxidase from *L. lactis*	100.1	1.82	89.9	This study

**Table 3:** Strains used in this study.

**Strain**	**Description**	**Genotype**	**Reference**
CEN.PK2-1C		*MAT***a** *ura3-52 trp1-289 leu2-3,112 his3*Δ*1 MAL2-8C SUC2*	EUROSCARF
*bdh1*Δ	BY4741 *bdh1*Δ	*MAT***a** *his3*Δ*1 leu2*Δ*0 met15*Δ*0 ura3*Δ*0 bdh1*Δ*::KanMX6*	EUROSCARF
JHY605	*adh1-5*Δ*gpd1*Δ*gpd2*Δ	CEN.PK2-1C *adh1*Δ*::loxP adh2*Δ*::loxP adh3*Δ*::loxP adh4*Δ*::loxP adh5*Δ*::loxP gpd1*Δ*::loxP gpd2*Δ*::loxP*	14
JHY617	*adh1-5*Δ*gpd1*Δ*gpd2*Δ*bdh1*Δ	CEN.PK2-1C *adh1*Δ*::loxP adh2*Δ*::loxP adh3*Δ*::loxP adh4*Δ*::loxP adh5*Δ*::loxP gpd1*Δ*::loxP gpd2*Δ*::loxP bdh1*Δ*::KanMX6*	This study
JHY605-C	*adh1-5*Δ*gpd1*Δ*gpd2*Δ [EV]	JHY605 harboring p413GPD	This study
JHY605-SD	*adh1-5*Δ*gpd1*Δ*gpd2*Δ [SD]	JHY605 harboring p413-SD	This study
JHY617-SD	*adh1-5*Δ*gpd1*Δ*gpd2*Δ*bdh1*Δ [SD]	JHY617 harboring p413-SD	This study
JHY617-SDN	*adh1-5*Δ*gpd1*Δ*gpd2*Δ*bdh1*Δ [SDN]	JHY617 harboring p413-SDN	This study

**Table 4:** Plasmids used in this study.

**Plasmid**	**Description**	**Reference**
p413GPD	CEN/ARS plasmid, *HIS3*, P_*TDH3,*_ T_*CYC1*_	29
p413-D	CEN/ARS plasmid, *HIS3,* P_*TEF1*_-*alsD*-T_*GPM1*_	14
p413-SD	CEN/ARS plasmid, *HIS3,* P_*TDH3*_-*alsS*-T_*CYC1*_, P_*TEF1*_-*alsD*-T_*GPM1*_	This study
p413-SDN	CEN/ARS plasmid, *HIS3*, P_*TDH3*_-*alsS*-T_*CYC1*_, P_*TEF1*_-*alsD*-T_*GPM1*_, P_*FBA1*_-*noxE*-T_*FBA1*_	This study
